# Routine intraoperative cholangiography during laparoscopic cholecystectomy: application of the 2016 WSES guidelines for predicting choledocholithiasis

**DOI:** 10.1007/s00464-021-08305-4

**Published:** 2021-02-01

**Authors:** Hui-Ying Lai, Kuei-Yen Tsai, Hsin-An Chen

**Affiliations:** 1 Graduate Institute of Clinical Medicine, College of Medicine, Taipei Medical University, Taipei, Taiwan; 2 Division of General Surgery, Department of Surgery, Taipei Medical University-Shuang Ho Hospital, Number 291, Zhongzheng Road, Zhonghe District, New Taipei City, 235 Taiwan; 3 Department of Surgery, School of Medicine, College of Medicine, Taipei Medical University, Taipei, Taiwan

**Keywords:** Intraoperative cholangiography, Laparoscopic cholecystectomy, Choledocholithiasis, Common bile duct stone, Bile duct injury

## Abstract

**Background:**

Routine use of intraoperative cholangiography (IOC) during laparoscopic cholecystectomy (LC) for detecting common bile duct stones remains controversial. The 2016 World Society of Emergency Surgery (WSES) guidelines on acute calculous cholecystitis proposed a risk stratification for choledocholithiasis. Our present study aimed to (1) examine the findings of common bile duct (CBD) stones in patients underwent LC with routine use of IOC, and (2) validate the 2016 WSES risk classes for predicting choledocholithiasis.

**Methods:**

All patients had LC with IOC routinely performed from November 2012 to December 2017 were reviewed retrospectively. Patients were classified into high-, intermediate-, and low-risk groups based on the 2016 WSES risk classes with modification.

**Results:**

A total of 990 patients with LC and routine IOC were enrolled. CBD stones were detected in 197 (19.9%) patients. The rate of CBD stone detected in low-, intermediate-, high-risk groups were 0%, 14.2%, and 89.6%, respectively. Predictors as following: evidence of CBD stones on abdominal ultrasound or computed tomography, CBD diameter > 6 mm, total bilirubin > 4 mg/dL, bilirubin level = 1.8–4 mg/dL, abnormal liver biochemical test result other than bilirubin, presence of clinical gallstone pancreatitis had statistical significance between patients with and without CBD stones. Major bile duct injury was found in 4 patients (0.4%). All 4 patients had uneventful recovery after repair surgery.

**Conclusions:**

Based on our study results, the 2016 WSES risk classes for choledocholithiasis could be an effective approach for predicting the risk of choledocholithiasis. Considering its advantages for detecting CBD stones and biliary injuries, the routine use of IOC is still suggested.

**Supplementary Information:**

The online version contains supplementary material available at 10.1007/s00464-021-08305-4.

Since its first introduction in the 1930s, intraoperative cholangiography (IOC) has been used to detect choledocholithiasis, biliary anatomy, and iatrogenic bile duct injuries. A prevalence of 10%–18% for concurrent common bile duct (CBD) stones in patients undergoing laparoscopic cholecystectomy (LC) has been reported, which may result in future health problems, such as pain, jaundice, infection, and biliary pancreatitis, if not managed in a timely manner [1, 2]. According to data from a large Swedish registry, when CBD stones are found on IOC during cholecystectomy, there is a higher rate of unfavorable outcomes if no measures are taken intraoperatively or planned postoperatively [3]. In addition, as LC being one of the most common abdominal operations performed currently, iatrogenic bile duct injury has become more frequent. In open cholecystectomy, the average incidence of CBD injury is approximately 0.2%–0.4%; however, in laparoscopic procedures, the incidence can increase to 0.2%–1.7%, especially in complicated cases [4, 5]. More biliary injuries can be detected intraoperatively when IOC is performed. Early detection and treatment of these injuries can prevent the further progression of partial injuries to complete CBD transections [6].

On the other hand, some stated that IOC increases the cost, operation time, and hospital stay, with no strong evidence indicating that it improves stone extraction and biliary injury rates [7]. When IOC was routinely performed, 10% of patients were found to have asymptomatic CBD stones, which may subsequently pass spontaneously or never cause any symptoms [8, 9].

So far, the need for routine IOC during LC remains controversial. As IOC is mostly performed when CBD stones are suspected, preoperative evaluation for the possibility of choledocholithiasis became a decisive factor. The 2016 World Society of Emergency Surgery (WSES) guidelines on acute calculous cholecystitis proposed a risk stratification for choledocholithiasis [10, 11]. Few studies have assessed the performance of existing guidelines for the prediction of choledocholithiasis [12, 13]. In these studies, CBD stones were diagnosed through endoscopic ultrasound (EUS), magnetic resonance cholangiopancreatography (MRCP), or endoscopic retrograde cholangiopancreatography (ERCP). Our present study examined the findings of CBD stones in laparoscopic cholecystectomy with routine intraoperative cholangiography, and aimed to (1) validate the 2016 WSES risk classes for predicting choledocholithiasis, (2) evaluate the performance of individual predictors, and (3) investigate the characteristics of patients susceptible to CBD stones. Cases with intraoperative bile duct injury were also recorded and further discussed.

## Materials and methods

We retrospectively reviewed the data of 990 patients who underwent elective and emergency LC with IOC routinely performed from November 2012 to December 2017. All surgeries were performed by a single surgical team. Some patients with jaundice or suspected CBD stones clinically had ERCP prior to operation. Patients with or without preoperative ERCP were included. Data of preoperative evaluations: imaging studies, biochemistry data; perioperative findings such as operation time, IOC completion, positive IOC findings, conversion to open surgery rate, and bile duct injury, length of hospital stay, readmission rate, and mortality and morbidity were collected. This study was approved by the ethics committee of our hospital.

### Operation method

A subumbilical incision was made through minilaparotomy to insert an 11-mm trocar, and insufflation of carbon dioxide was maintained at a pressure of 15 mm Hg. Surgery was performed using a 30° laparoscope. Another incision was made at the subxiphoid to insert another 11-mm trocar. In addition, two other incisions were performed at the midclavicular and right subcostal for inserting 5-mm trocars for placing two clamps to hold the gallbladder on its fundus and Hartmann pouch. L-hook electrocautery was applied for dissection.

After the cystic duct had been identified, a metallic clip was placed between the cystic duct and gallbladder neck. Subsequently, a small hole was created in the cystic duct with a laparoscopic scissor. An IOC catheter was inserted into the cystic duct and fixed with another clip. The biliary anatomy was visualized with a mobile C-arm. After no filling defect was found in the bile duct with adequate flow of contrast medium into the duodenum, the IOC catheter was removed, and operation was completed following the common procedure. Otherwise, laparoscopic common bile duct exploration (LCBDE) or postoperative ERCP was performed.

### Risk classes

Patients were classified into high-, intermediate-, and low-risk groups based on the 2016 WSES risk classes with modification. Predictive factors included the following: preoperative abdominal ultrasound (AUS) or computed tomography (CT), bilirubin, liver function test, age, and gall stone pancreatitis clinically. Patients had high risk if they had evidence of CBD stones on AUS or CT; patients had intermediate risk if they had at least one of the other risk factors (CBD diameter > 6 mm, total bilirubin > 4 mg/dL, bilirubin level = 1.8–4 mg/dL, abnormal liver biochemical test result other than bilirubin, age > 55 years, and presence of clinical gallstone pancreatitis); and patients with none of these risk factors were classified into the low-risk group (Table 1).

**Table 1:** Predictive factors and risk classes for choledocholithiasis

Very strong	Evidence of CBD stones on AUS or CT**
*Predictive factors for choledocholithiasis*	
Strong	CBD diameter > 6 mm (with gallbladder in situ)
	Total bilirubin > 4 mg/dL
	Bilirubin level: 1.8–4 mg/dL
Moderate	Abnormal liver biochemical test other than bilirubin
	Age > 55 years
	Clinical gallstone pancreatitis
*Risk classes for choledocholithiasis*	
High	Presence of any VERY STRONG predictors
Low	No predictors present
Intermediate	All other patients

Modified from [10, 11]

**In “very strong predictor,” this study also included CT as a diagnostic tool

### Statistical analysis

Validity of the WSES classification for detecting choledocholithiasis and performance of individual risk factors were examined, and basic characteristics were compared between patients with or without CBD stones. Among patients receiving ERCP prior to LC, those with positive IOC findings during operations were further analyzed.

Baseline characteristics and predictive factors were compared using Student’s *t* test for continuous variables and Chi-square and Fisher’s exact test for categorical variables, as appropriate. A p value of < 0.05 was considered statistically significant. Individual predictor cut-off points were estimated using the receiver operator characteristic (ROC) curve. Statistical analysis was conducted using IBM SPSS Statistics for Windows, Version 25.0. Armonk, NY: IBM Corp.

## Results

A total of 990 patients with LC and routine IOC were enrolled into this study. The study population comprised 498 male and 492 female patients (M:F ratio = 1:1). The mean age of our patients was 58.65 (± 16.12) years. Patients were admitted mainly for gallbladder stones (90%), and they had other surgery indications, including gallbladder tumor/polyps and CBD stones. Moreover, 131 patients had previous ERCP; 833 patients had elective surgery, and the remaining 157 had emergent surgery.

In this study, 128 patients had positive IOC findings; 120 had direct LCBDE, 4 had direct LCBDE with postoperative ERCP, 3 had postoperative ERCP (LCBDE failed due to the small caliber of CBD), and the stone was passed in another 1 patient after pushing contrast medium. Postoperative ERCP after direct LCBDE was arranged in 3 patients for large impacted stones that could not be removed, and in 1 patient, the contrast medium did not pass smoothly, although no filling defect was detected on IOC after LCBDE. In 33 patients, IOC was not performed successfully mostly because of the small caliber of the cystic duct or fragile tissue. In 11 patients, laparoscopic operations were converted to open surgery. The average operation time was 78.20 ± 35.34 min. The mean length of hospital stay was 3.06 ± 3.21 days.

Two patients died (1 had respiratory failure, and the other had postoperative bile leak with septic shock). Major bile duct injury was found in 4 patients. Immediate primary repairs were performed in 2 patients, and 1 patient underwent hepaticojejunostomy (HJ) after conversion into laparotomy. Another patient was readmitted on POD 8 and underwent primary repair through laparoscopy. The patient who underwent HJ had low risk, and other 3 patients had intermediate risk. All 4 patients had a smooth hospital course. Minor bile leak (defined as bile content in drainage found postoperatively) was found in 5 patients, and 2 patients had postoperative endoscopic retrograde biliary drainage (ERBD), whereas 3 patients recovered without specific intervention. Other postoperative complications were noted in 19 patients: respiratory-related complications in 5, intra-abdominal infection in 3, postoperative ileus in 2, wound infection in 2, iatrogenic small bowel perforation in 2, cardiovascular-related complication in 1, cholangitis in 1, urinary tract infection in 1, duodenum bleeding in 1, and stroke in 1 patient. Moreover, 36 patients were readmitted after operation, including 9 for CBD stones (Table 2).

**Table 2:** Perioperative findings and postoperative outcomes (*N* = 990)

	*n*
Positive IOC findings	128 (12.9%)*
LCBDE	120 (93.8%)
LCBDE → ERCP	4 (3.1%)
ERCP	3 (2.3%)
IOC failed	33 (3.3%)
Conversion to open	11 (1.1%)
OP time (minutes)	78.20 ± 35.34
Main bile duct injury	4 (0.4%)
Primary repair	3
Hepaticojejunostomy	1
Postoperative complications	19 (1.9%)
Minor bile leak	5
Length of stay (days)	3.05 ± 3.21
Readmission	36 (3.6%)
For CBD stone	9
Mortality	2 (0.2%)

*One CBD stone was flushed out after pushing contrast medium

CBD stones were detected in 197 (19.9%) patients. After applying the choledocholithiasis risk classification to our 990 patients, 318, 537, and 135 patients were included in the low-risk, intermediate-risk, and high-risk groups, respectively; the rate of CBD stone detection was 0%, 14.15%, and 89.63% in these groups, respectively. In the intermediate and high-risk groups, the risk classification had a sensitivity of 100% and specificity of 40.10% (accuracy: 52.02%) (Table 3).

**Table 3:** CBD stone detection performance

	All *N* = 990	Low risk *n* = 318	Intermediate risk *n* = 537	High risk *n* = 135
ERCP first	131	0	47	84
Positive findings on ERCP	103	0	40	63
Positive findings on IOC	57	0	11	46
Both	35	0	7	28
Direct LC	859	318	490	51
Positive findings on IOC	72	0	32	40
Numbers of CBD stone detected	197	0	76	121
CBD stone detection rate	19.9%	0%	14.2%	89.6%
Sensitivity	/	/	100%	
Specificity	/	/	40.10%	

Basic characteristics, imaging studies, and biochemical variables of patients with and without CBD stones are presented in Table 4. Among these parameters, statistically significant differences were found in age, total bilirubin, GOT, GPT, and CBD diameter (> 6 mm) between patients with and without CBD stones. Regarding the performance of individual predictors (Table 5), all these predictors, except “age > 55 years”, had statistical significance between patients with and without CBD stones. Further ROC curve analysis of age showed the optimal cut-off point to be 63.5 years old.

**Table 4:** Baseline characteristic comparisons of patients with/without CBD stones

	All *N* = 990	CBD stone ( +) *n* = 197	CBD stone ( −) *n* = 793	*p* value
Age	56.28 ± 15.40	59.94 ± 16.84	55.26 ± 14.84	0.012*
Female	492	99	393	0.525
BMI	25.71 ± 5.07	25.07 ± 4.41	25.89 ± 5.23	0.187
WBC	8.69 ± 5.42	9.14 ± 4.07	8.56 ± 5.73	0.387
Bil-T	1.29 ± 2.15	2.79 ± 3.94	0.87 ± 0.90	< 0.001*
GOT	68.12 ± 115.72	165.71 ± 185.72	41.05 ± 65.14	< 0.001*
GPT	69.02 ± 119.97	177.25 ± 203.94	40.31 ± 58.06	< 0.001*
CBD diameter > 6 mm	208	163	45	< 0.001*

*Statistically significant (*p* < 0.05)

**Table 5:** Individual risk predictor performance

	All *N* = 990	CBD stone ( +) *n* = 197	CBD stone ( −) n = 793	*p* value
Very strong predictor				
CBD stones on CT/AUS	135 (13.64%)	115 (58.97%)	20 (2.52%)	< 0.001*
Strong predictor				
CBD > 6 mm	160 (16.16%)	114 (58.16%)	46 (5.79%)	< 0.001*
Total bilirubin > 4 mg/dL	37 (3.7%)	34 (17.35%)	3 (0.04%)	< 0.001*
Bilirubin 1.8–4 mg/dL	112 (11.31%)	63 (31.63%)	49 (6.17%)	< 0.001*
Moderate predictor				
Abnormal liver biochemistry data	349 (35.25%)	157 (80.10%)	192 (24.18%)	< 0.001*
Age > 55 years	486 (49.09%)	109 (55.61%)	377 (47.48%)	0.168
Clinical gallstone pancreatitis	33 (3.33%)	30 (15.31%)	3 (0.38%)	< 0.001*

*Statistically significant (*p* < 0.05)

In this study, 131 patients received ERCP before LC, and 72 patients had successful stone retrieval. IOC was not conducted successfully in 2 of the 72 patients. For 70 patients who underwent subsequent LC with IOC, 10 patients had still had positive IOC findings. None of the patient characteristics showed significant differences between patients with and without residual CBD stone during IOC (Table 6).

**Table 6:** Comparison of IOC findings (+ / −) after ERCP stone retrieval (*N* = 72)

	→IOC (−) *n* = 60	→IOC (+) *n* = 10 (13.9%)	*p* value
Age	57.38 ± 16.27	66.20 ± 17.05	0.120
Female	30	6	0.736
BMI	25.42 ± 4.66	23.66 ± 2.86	0.252
Bil-T	0.96 ± 0.52	1.15 ± 1.19	0.627
GOT	48.02 ± 50.36	78.70 ± 156.67	0.554
GPT	42.89 ± 52.29	79.70 ± 119.43	0.361

IOC failed in 2 patients

Biochemistry laboratory data were collected before LC

## Discussion

In our study, 859 patients underwent direct LC, and approximately 13% of the study patients had positive IOC findings, similar to the results of other studies [14–16]. After applying the risk classification (Table 1), no CBD stones were detected among low-risk patients. This result suggests that there is no need for patients with none of the predictors to undergo IOC for detecting CBD stones. Although this risk classification showed low specificity and accuracy among intermediate and high-risk groups (sensitivity: 100%; specificity: 40.10%; accuracy: 52.02%), CBD stones were still found in almost 15% of the patients in the intermediate-risk group. Tabone et al. reported a case series with the selective use of IOC in 266 patients in 1308 cholecystectomies. CBD stones were found in 13.5% of patients who underwent IOC, and 72% of them had normal preoperative images [17]. Regarding further interventions to clear the bile duct are generally suggested, routine IOC should be considered in patients with intermediate risk.

A previous study had used similar risk classification for choledocholithiasis to verify the performance of individual predictors. It showed statistically significant differences in factors including CBD stones on abdominal US, ascending cholangitis, CBD diameter of > 6 mm on US, and gallstone pancreatitis. However, such predictors did not increase the accuracy or performance of risk classes [13]. Our study also found significant differences in all predictors, except for age. The ROC curve was then calculated, and a cut-off point of 63.5 years was found for age. However, as we re-sorted our study group, we found that most intermediate-risk patients had more than one risk predictor. In other words, our elderly patients often presented with both elevated liver function and serum bilirubin levels. Boland et al. and Cieslak et al. have noted higher liver function data in the elderly population [18, 19]. Thus, correlations between individual risk predictors need to be further clarified to determine the most precise evaluation tools.

In the subgroup of 72 patients who had previous ERCP for stone retrieval, CBD stones were still found on IOC in 10 patients (13.9%). However, no significant decisive predictors were detected in the subgroup. In a study of 345 patients who underwent endoscopic biliary sphincterotomy for CBD stone recurrence, patient-related risk factors, such as periampullary diverticulum, CBD diameter (> 15 vs. ≤ 15 mm), and quantity of stones, as well as procedure-related risk factors, including complete stone removal in the first session and lithotripsy, were associated with CBD stone recurrence [20].

In cases with persistent stone on patients who underwent ERCP before cholecystectomy, we tend to perform direct LCBDE if the CBD diameter is ≥ 8 mm. Although postoperative ERCP is also feasible, single-stage management shows advantages in terms of a lower rate of technique failure, a fewer number of procedures, shorter hospital stay, and lower hospital charges [21, 22]. Also, if patients underwent LCBDE were found to have multiple CBD stones, have a clinical suspicion of residual stones or further stone formation, T-tube drainage would be used. With the T-tube, post-op diagnostic and therapeutic choledochoscopy could also be performed. In our practice, a planned postoperative ERCP was mostly reserved for patients with a small diameter of CBD to prevent biliary stenosis after LCBDE.

In the present study, main biliary duct injury was found in 4 patients (0.4%), and immediate repairs were performed during operation in 3 patients. These injuries were small perforation of the main bile ducts, and no transection injury was found. In a population-based cohort study in Sweden, Törnqvist et al. reported a rate of 1.5% for iatrogenic bile duct injuries among 51,041 cholecystectomies. In that study, patients with early detection of a bile duct injury (during the primary operation) showed a higher survival rate than those with delayed detection. Moreover, the intention to perform IOC also reduced the risk of death [23]. Without IOC, intraoperative diagnosis of biliary injury was made in only 10%–20% of the patients. By contrast, 30–90% of the injury could be detected during operation when IOC was used, and most injuries could be repaired immediately, with satisfactory outcomes. Although IOC may not prevent all biliary injuries, routine application reduces the rate and severity of CBD injury [24–26].

Given the rarity of CBD injury and the actual risk reduction associated with IOC, it has been argued that its routine application does not justify the increased time and cost. Flum et al. showed that routine IOC prevents 2.5 deaths for every 10,000 patients [26]. Ludwig et al. also reported that IOC can prevent CBD injury per 500–700 cholecystectomies, and the surgical management costs for a CBD injury are $8000–20,000 [27]. The cost of undetected biliary injury with repair done in the late course is 16 times higher than the cost of the injury with immediate repair. Moreover, potential psychological, biological, and socially effects on patients with biliary injury should also be considered [28]. Because no accurate predictors or characteristics are available for identifying patients prone to bile duct injury, routine IOC may remain a cost-effective and safe approach [25].

### Limitation

This study is limited by the fact that routine IOC was not compared with selective use. Correlations between each variable should also be considered. A randomized control study should be conducted.

### Conclusion

Based on our study results, the 2016 WSES risk classes for choledocholithiasis could be an effective approach for predicting the risk of CBD stones. No CBD stone was detected in our 318 low-risk patients; yet CBD stones were found in 14.2% of the patients in the intermediate-risk group. Our results also indicate that despite undergoing preoperative ERCP, IOC should still be performed. Additionally, according to a literature review, IOC enables the early detection of bile duct injury, which facilitates more favorable survival and prognosis for patients. Considering its advantages for detecting CBD stones and biliary injuries, the routine use of IOC is still suggested.

## Supplementary Information

Below is the link to the electronic supplementary material.

Supplementary file1 (docx 15 KB)

Supplementary file2 (docx 16 KB)
