# Supplementary material for: Routine intraoperative cholangiography during laparoscopic cholecystectomy: application of the 2016 WSES guidelines for predicting choledocholithiasis
Source: Surg Endosc. 2021 Feb 1;36(1):461–7. doi: 10.1007/s00464-021-08305-4 (PMC8741698; doi:10.1007/s00464-021-08305-4)
Supplement: Supplementary file 1 — Supplementary file1 (docx 15 KB) [file 464_2021_8305_MOESM1_ESM.docx]

Dear editor,

I am writing to submit our manuscript entitled, “Routine intraoperative cholangiography during laparoscopic cholecystectomy: Application of the 2016 WSES guidelines for predicting choledocholithiasis”, for consideration of publication in Surgical Endoscopy. This paper has been accepted by the EAES congress 2020.

There have been discussions on the accuracy of pre-operative predictive factors for the presence of common bile stone, and the necessity of routine use of intraoperative cholangiography during laparoscopic cholecystectomy. However, most studies validated the presence of choledocholithiasis by endoscopic ultrasound, magnetic resonance cholangiopancreatography, or more invasively, endoscopic retrograde cholangiopancreatography. In our study, we demonstrate our experiences on a 5-year-period consecutive 990 LCs with routine IOC with one single surgical team. Application of the newest guideline and further discussion are also presented. All authors approved the manuscript and the submission. Thank you for receiving our manuscript and considering it for reviewing. We appreciate your time and look forward to your response.

Kind regards,

Hui-Ying, Lai

General Surgery

Shuang Ho Hospital

New Taipei City, Taiwan
