# Supplementary material for: Routine intraoperative cholangiography during laparoscopic cholecystectomy: application of the 2016 WSES guidelines for predicting choledocholithiasis
Source: Surg Endosc. 2021 Feb 1;36(1):461–7. doi: 10.1007/s00464-021-08305-4 (PMC8741698; doi:10.1007/s00464-021-08305-4)
Supplement: Supplementary file 2 — Supplementary file2 (docx 16 KB) [file 464_2021_8305_MOESM2_ESM.docx]

Dear Editor,

We would like to express our sincere gratitude to you and the reviewers for the positive feedback and helpful comment on our submission: *Routine intraoperative cholangiography during laparoscopic cholecystectomy: Application of the 2016 WSES guidelines for predicting choledocholithiasis* to Surgical Endoscopy. We have revised our paper, and the revised portion are marked in red in the paper. These additional discussions have helped us to substantially improve our manuscript.

On the following pages, we outline point by point responses to the comments by the reviewers. We hope our revised version will be received favorably and look forward to hearing from you soon.

*Reviewer #1: Very good trial, which should be published. This study is limited by the fact that routine IOC was not compared with elective use. Correlations between each variable should also be considered. A randomized control study should be conducted.*

Thank you very much for this comment. We do agree that comparisons between elective use of IOC are worthy of further analysis as mentioned in the “limitation” part. And we are already planning future studies.

*Reviewer #2: Thank you for a thorough study on an ongoing attempt to reduce bile duct injury. The premise of the paper is well defined. The conclusion is applicable in current clinical setting. Please address the following questions if all possible in your paper.*

***In the US, majority of cholecystectomies are performed in outpatient setting. Can you discuss the practice in Taiwan that may contribute to an average hospitalization of 3+/- 3 days?*

We genuinely appreciate your kind feedback.

In Taiwan, most of the cost for cholecystectomy and hospitalization is covered by Taiwan National Health Insurance (NHI). According to the NHI payment systems, it is mandatory for patients undergoing general anesthesia to be admitted. Besides, this study included patients who underwent emergent surgeries. In cases with severe inflammations or bleeding tendency, we sometimes place a subhepatic drainage tube to monitor post-op conditions. Nearly all the drains are removed before discharge. As a result, an average hospitalization of 3 days is presented in our study.

***From your analysis of persistent stone found for patients who underwent ERCP prior to cholecystectomy (1/7 patients), would you recommend reserving ERCP after cholecystectomy for your patient population?*

We have added one paragraph to the manuscript which presents our management on these conditions. (Discussion- paragraph 4^th^)

In cases with persistent stone on patients who underwent ERCP before cholecystectomy, we tend to perform direct LCBDE if the CBD diameter is >= 8mm. Although postoperative ERCP is also feasible, single-stage management shows advantages in terms of a lower rate of technique failure, a fewer number of procedures, shorter hospital stay, and lower hospital charges. Also, if patients underwent LCBDE were found to have multiple CBD stones, have a clinical suspicion of residual stones or further stone formation, T-tube drainage would be used. With the T-tube, post-op diagnostic and therapeutic choledochoscopy could also be performed. In our practice, a planned post-operative ERCP was mostly reserved for patients with a small diameter of CBD to prevent biliary stenosis after LCBDE.

Thank you once again for your time and interest.

Regards,

Hui-Ying, Lai

General Surgery, Shuang Ho Hospital, New Taipei City, Taiwan
